# Light at night and circadian rhythms: from the perspective of physiological anthropology research

**DOI:** 10.1186/s40101-024-00380-5

**Published:** 2024-12-26

**Authors:** Shigekazu Higuchi

**Affiliations:** Department of Human Life Design and Science, Faculty of Design, Kyushu University, 4-9-1 Shiobaru, Minami-Ku, Fukuoka City, Fukuoka, Japan

Physiological anthropology focuses on human adaptations to the environment and their variations. It is characterised by the study of human adaptation to various artificial environments created by modern society such as artificial light. Artificial light at night (LAN) is an indispensable component of modern society. However, it negatively affects circadian rhythms and sleep [1]. The adverse effects of LAN on various animals and plants are termed ‘light pollution’. Throughout the long history of human evolution, our ancestors would not have experienced nights as bright as those of the modern society. Today’s bright nights may represent an abnormal environment in the context of human history. Determining whether humans can adapt to LAN and the effects of LAN are important research topics in physiological anthropology [2, 3].

Light entering the eye is transmitted via the retinohypothalamic tract to the suprachiasmatic nucleus, which is the centre of the circadian clock. LAN can delay the circadian rhythm phase by shifting the internal circadian clock to a nocturnal type. It also inhibits the secretion of melatonin, which is responsible for informing the human body about nighttime. In addition, light inhibits the increase in natural nighttime sleepiness and decrease in body temperature. These effects are referred to as non-visual effects of light. The effects of light depend on the intensity, wavelength, time of exposure, and duration of exposure [1]. A particularly significant event is the discovery of new photosensitive cells in the retina other than rods and cones. These cells, called intrinsically photosensitive retinal ganglion cells (ipRGCs), contain a photosensitive protein, melanopsin, and are highly responsive to blue light near 480 nm, which is important for the non-visual effects of light.

However, individual differences exist in susceptibility to light [4] owing to individual differences in photosensitivity of the circadian system. For example, photosensitivity differs between different ages, with children being more sensitive to light than adults [5]. Furthermore, Europeans are more sensitive to light than East Asians [6]. There are also differences in light sensitivity among individuals of the same age. Genetic factors and history of light exposure may cause individual differences. Therefore, studies on shift workers are also needed because their occupations are more susceptible to the effects of LAN.

A recent study reported recommendations for LAN to prevent adverse effects on sleep and circadian rhythms [7]. In the study, room lighting is recommended to be maintained below 10 melanopic equivalent daylight illuminance (EDI; lx) for 3 h before bedtime (vertical illuminance at 1.2 m above the floor). Melanopic EDI is a unit of stimulation for ipRGCs but has recently been introduced as a unit to indicate the non-visual effects of light [8]. For example, the recommended 10 melanopic EDI is approximately 25 lx illuminance at a colour temperature of 3000 K and approximately 12 lx at a colour temperature of 6000 K. However, studies examining light exposure among the Japanese and Chinese suggest that few people spend the night in rooms with such low illuminance [9]. In addition to cultural factors, this may be related to ethnic differences in light sensitivity. Future studies on these recommendations should consider the diversity of light sensitivity, including ethnic and age-related differences.

To date, most studies on the effects of light have been conducted in Europe and North America. However, considering the use of artificial lighting in all regions worldwide, research on diverse ethnic groups is necessary from an anthropological perspective. The Western, Educated, Industrialised, Rich, and Democratic (WEIRD) issue has been proposed as a bias arising from subject selection in academic research [10]. Similar findings have been noted in studies of the non-visual effects of light [11]. Future research is increasingly important to understand the diversity of light sensitivity. To assess the diversity of light effects, it is necessary to comprehensively describe the conditions under which light exposure occurs. This will make it easier to compare the results of studies conducted in different regions and laboratories. A consensus report has been published summarising expert opinions on items that would be desirable to describe [12]. Studies submitted to the *Journal of Physiological Anthropology* should also conform to these guidelines.

In recent years, there has been an increase in research on non-visual effects of light in the *Journal of Physiological Anthropology* [9, 13–16], which involves both laboratory experiments and field studies. In addition to nighttime light, daylight is important for humans and associated with myopia [17, 18]. In addition to light environments, there are significant researches on sleep and circadian rhythm under various environments, including thermal [19–21], nutrition [22, 23], and social environments [24]. There are significant researches in children [13, 14, 21, 25]. In the future, additional research from various perspectives, including the exploration of interactions with these environments and ages, is expected to clarify the adaptation to the light environment and its diversity.
